# Supplementary material for: A scoping review on peer-led interventions to improve youth mental health in low- and middle-income countries
Source: Glob Ment Health (Camb). 2024 Dec 16;12:e1. doi: 10.1017/gmh.2024.149 (PMC11704389; doi:10.1017/gmh.2024.149)
Supplement: Chow et al. supplementary material [file S2054425124001493sup001.docx]

# **Supplementary Materials**

## **Search Strategies**

**Librarian Searcher: Beth Blackwood, MSLS; Duke University Medical Center Library & Archives, Duke University School of Medicine**

**Peer review of search conducted by:** Sarah Cantrell, MLIS; Duke University Medical Center Library & Archives, Duke University School of Medicine

**Database:** MEDLINE (via Ovid)
**Search Date**: 9/6/2024

| **Concept** | **Strategy** | **Results** |
| --- | --- | --- |
| 1 Adolescents | exp adolescent/ OR exp young adult/ or (young adult or youth or youths or adolescent or adolescents or teen or teens or teenager or teenagers or teenaged or high school or high schools or high schooler or high schoolers or secondary school or secondary schools or secondary schooler or secondary schoolers or college or pubescent or pubescence or young person or young persons or young people or puberty).ti,ab. not ((exp adult/ OR exp infant/) not exp adolescent/) | 2,500,105 |
| 2 Mental Health | exp mental health/ or exp mental disorders/ or exp suicide/ or exp anxiety/ or exp anxiety disorders/ or exp disruptive, impulse control, and conduct disorders/ or exp dissociative disorders/ or exp mood disorders/ or exp depressive disorder/ or exp neurocognitive disorders/ or exp cognition disorders/ or exp schizophrenia spectrum and other psychotic disorders/ or exp behavioral symptoms/ or exp cognition/ or dyssomnias/ or exp stress disorders, post-traumatic/ or ((behavior* or behaviour* or mental or mood or psychologic* or psychiatric or neurocogn* or neuropsychiatric or neuro-psychological or neuro-psychiatric or neuropsychological or mental or mentally or stress* or affective or panic* or phobic or phobia* or psychotic or somatoform) adj3 (health or disease or diseases or disorder or disorders or illness or ill or symptom or symptoms or symptomatic or syndrome or syndromes or ailment or ailments)).ti,ab. or (suicid* or depress* or PTSD or CPTSD or anxiety or anxious).ti,ab. | 1,664,661 |
| 3 Peer Support | exp peer groups/ or exp psychotherapy, group/ or ((peer or peers) adj2 (group* or support* or educat* or instruct* or facilitat* or mentor* or influenc* or navigat* or leader* or led or supervis* or manag* or relation* or communicat* or relationship* or influence* or relationship* communit* or network*)).ti,ab. or (peer?peer or "peer to peer" or "peer-to-peer" or peer-group or peer-led or peer-mentor*).ti,ab. or ("group interpersonal psychotherapy" or "group therapy" or group-therapy).ti,ab. | 75,936 |
| 4 LMIC Mesh | exp "developing countries"/ or exp afghanistan/ or exp bangladesh/ or exp benin/ or exp "burkina faso"/ or exp burundi/ or exp cambodia/ or exp "central african republic"/ or exp chad/ or exp comoros/ or exp "democratic republic of the congo"/ or exp eritrea/ or exp ethiopia/ or exp gambia/ or exp guinea/ or exp guinea-bissau/ or exp haiti/ or exp kenya/ or exp "democratic people's republic of korea"/ or exp liberia/ or exp madagascar/ or exp malawi/ or exp mali/ or exp mozambique/ or exp myanmar/ or exp nepal/ or exp niger/ or exp rwanda/ or exp "sierra leone"/ or exp somalia/ or exp tajikistan/ or exp tanzania/ or exp togo/ or exp uganda/ or exp zimbabwe/ or exp armenia/ or exp bhutan/ or exp bolivia/ or exp cameroon/ or exp "cabo verde"/ or exp congo/ or exp "cote d'ivoire"/ or exp djibouti/ or exp egypt/ or exp "el salvador"/ or exp "georgia (republic)"/ or exp ghana/ or exp guatemala/ or exp guyana/ or exp honduras/ or exp indonesia/ or exp india/ or exp kosovo/ or exp kyrgyzstan/ or exp laos/ or exp lesotho/ or exp mauritania/ or exp micronesia/ or exp moldova/ or exp mongolia/ or exp morocco/ or exp nicaragua/ or exp nigeria/ or exp pakistan/ or exp "papua new guinea"/ or exp paraguay/ or exp philippines/ or exp "independent state of samoa"/ or exp "atlantic islands"/ or exp senegal/ or exp melanesia/ or exp "sri lanka"/ or exp sudan/ or exp "south sudan"/ or exp eswatini/ or exp syria/ or exp timor-leste/ or exp ukraine/ or exp uzbekistan/ or exp vanuatu/ or exp vietnam/ or exp "middle east"/ or exp yemen/ or exp zambia/ or exp angola/ or exp albania/ or exp algeria/ or exp "american samoa"/ or exp argentina/ or exp azerbaijan/ or exp "republic of belarus"/ or exp belize/ or exp "bosnia and herzegovina"/ or exp botswana/ or exp brazil/ or exp bulgaria/ or exp china/ or exp colombia/ or exp "costa rica"/ or exp cuba/ or exp dominica/ or exp "dominican republic"/ or exp ecuador/ or exp "equatorial guinea"/ or exp fiji/ or exp gabon/ or exp grenada/ or exp iran/ or exp iraq/ or exp jamaica/ or exp jordan/ or exp kazakhstan/ or exp lebanon/ or exp libya/ or exp "republic of north macedonia"/ or exp malaysia/ or exp "indian ocean islands"/ or exp mexico/ or exp montenegro/ or exp namibia/ or exp palau/ or exp panama/ or exp peru/ or exp romania/ or exp russia/ or exp serbia/ or exp seychelles/ or exp "south africa"/ or exp "saint lucia"/ or exp "saint vincent and the grenadines"/ or exp suriname/ or exp thailand/ or exp tonga/ or exp tunisia/ or exp turkey/ or exp turkmenistan/ or exp venezuela/ | 1,430,211 |
| 5 LMIC Terms + Demonyms | (afghanistan OR afghan* OR afghanistani* OR afghani* OR afghanese OR bangladesh OR bangladeshi* OR benin OR 'edo people' OR 'edo person' OR bini OR 'burkina faso' OR burkinabe* OR burundi OR burundian* OR cambodia OR cambodian* OR 'cabo verde' OR 'cape verdean' OR 'cape verdeans' OR 'cabo verdean' OR 'cabo verdeans' OR 'central african republic' OR 'central african' OR 'central africans' OR chad OR chadian* OR comoros OR comorian* OR 'democratic republic of the congo' OR congolese* OR eritrea OR eritrean* OR ethiopia OR ethiopian* OR gambia OR gambian* OR guinea OR guinean* OR guinea-bissau OR 'bissau guinean' OR 'bissau guineans' OR bissau-guinean* OR haiti OR haitian* OR kenya OR kenyan* OR 'democratic peoples republic of korea' OR 'north korean' OR 'north koreans' OR liberia OR liberian* OR madagascar OR malagasy* OR madagascan* OR malawi OR malawian* OR mali OR malian* OR mozambique OR mozambican* OR myanmar OR burmese* OR myanma* OR nepal OR nepali* OR nepalese OR niger OR nigerien* OR rwanda OR rwandan* OR rwandese OR 'sierra leone' OR 'sierra leonean' OR 'sierra leoneans' OR somalia OR somali* OR tajikistan OR tajikstani* OR tajik* OR tanzania OR tanzanian* OR togo OR togolese* OR uganda OR ugandan* OR zimbabwe OR zimbabwean* OR zimbo* OR armenia OR armenian* OR bhutan OR bhutanese OR bolivia OR bolivian* OR cameroon OR cameroonian* OR 'cape verde' OR congo OR 'cote d ivoire' OR ivorian* OR djibouti OR djiboutian* OR egypt OR egyptian* OR 'el salvador' OR salvadoran* OR (georgia adj2 Republic) OR georgian* OR ghana OR ghanaian* OR guatemala OR guatemalan* OR guatemalteco* OR guatemalense* OR guyana OR guyanese OR honduras OR honduran* OR indonesia OR indonesian* OR india OR indian* OR kiribati OR gilbertese* OR kosovo OR kosovar* OR kosovan* OR kyrgyzstan OR kyrgyzstani* OR kirgiz OR kirghiz OR kyrgyz OR laos OR laotian* OR lao OR lesotho OR mosotho* OR basotho* OR mauritania OR mauritanian* OR micronesia OR micronesian* OR moldova OR moldovan* OR mongolia OR mongolian* OR morocco OR moroccan* OR nicaragua OR nicaraguan* OR nigeria OR nigerian* OR pakistan OR pakistani* OR 'papua new guinea' OR 'papua new guinean' OR 'papua new guineans' OR paraguay OR paraguayan* OR philippines OR filipin* OR pinoy* OR pinay* OR 'independent state of samoa' OR samoan* OR 'atlantic islands' OR 'sao tome' OR 'sao tomean' OR 'sao tomeans' OR santomean* OR principe OR senegal OR senegalese* OR melanesia OR melanesian* OR 'solomon islands' OR 'solomon islander' OR 'solomon islanders' OR 'sri lanka' OR 'sri lankan' OR 'sri lankans' OR sinhalese OR sudan OR sudanese OR swaziland OR swazi* OR liswati* OR eswatini OR syria OR syrian* OR 'east timor' OR 'east timorese' OR 'timor leste' OR timorese OR ukraine OR ukrainian* OR uzbekistan OR uzbekistani* OR vanuatu OR vanuatuan* OR vietnam OR vietnamese* OR 'middle east' OR 'middle eastern' OR 'west bank' OR gaza OR palestinian* OR gazan* OR yemen OR yemeni* OR zambia OR zambian* OR angola OR angolan* OR albania OR albanian* OR algeria OR algerian* OR argentina OR argentine* OR argentinean* OR argentinian* OR samoa OR samoan* OR azerbaijan OR azerbaijani* OR azeri* OR 'republic of belarus' OR belarus OR belarusian* OR belize OR belizean* OR bosnia-herzegovina OR bosnian* OR botswana OR batswana* OR motswana* OR brazil OR brazilian* OR bulgaria OR bulgarian* OR china OR chinese OR colombia OR colombian* OR 'costa rica' OR 'costa rican' OR 'costa ricans' OR cuba OR cuban* OR dominica OR dominican* OR 'dominican republic' OR ecuador OR ecuadorian* OR 'equatorial guinea' OR equatoguinean* OR 'equatorial guinean' OR 'equatorial guineans' OR fiji OR fijian* OR gabon OR gabonese OR gabonaise OR grenada OR grenadian* OR iran OR iranian* OR iraq OR iraqi* OR jamaica OR jamaican* OR jordan OR jordanian* OR kazakhstan OR kazakhstani* OR lebanon OR lebanese OR libya OR libyan* OR macedonia OR macedonian* OR malaysia OR malaysian* OR 'indian ocean islands' OR maldives OR maldivian* OR 'marshall islands' OR marshallese OR mauritius OR mauritian* OR mexico OR mexican* OR montenegro OR montenegrin* OR namibia OR namibian* OR palau OR palauan* OR panama OR panamanian* OR peru OR peruvian* OR romania OR romanian* OR russia OR russian* OR serbia OR serbian* OR seychelles OR seychellois* OR seselwa* OR 'south africa' OR 'south african' OR 'south africans' OR 'saint lucia' OR 'saint lucian' OR 'saint lucians' OR 'st vincent' OR 'saint vincent' OR grenadines OR 'west indies' OR vincentian* OR grenadinian* OR vincy OR suriname OR surinamese* OR thailand OR thai OR tonga OR tongan* OR tunisia OR tunisian* OR turkey OR turkish OR turk OR turkmenistan OR turkmenistani* OR turkmen* OR turkmenian* OR tuvalu OR tuvaluan* OR venezuela OR venezuelan*).ti,ab,kf,kw. | 2,134,155 |
| 6 LMIC Additional Terms | ("low resource" or "under-resourced" or underresourced or "resource poor" or "resource limited" or "under-developed" or underdeveloped or "developing country" or "developing countries" or "developing world" or "third world" or lmic or lmics or "central America" or "central american" or "south America" or "south American" or "southeast Asia" or "southeast Asian" or "pacific islands" or "pacific islander" or "pacific islanders" or sub-saharan or Caribbean or "latin America" or "latin American" or oceania).ti,ab,kf,kw. or (low adj2 income).ti,ab,kf,kw. or (middle adj2 income).ti,ab,kf,kw. or (low- adj2 income).ti,ab,kf,kw. or (middle- adj2 income).ti,ab,kf,kw. | 366,164 |
| 7 Combined LMIC Filter | 4 OR 5 OR 6 | 2,702,241 |
| 8 Combined | 1 AND 2 AND 3 AND 7 | 1,292 |
| 9 Exclusions | 8 not (case reports OR editorial OR letter OR comment).pt. | 1,270 |
| Validation String | **34292429 OR 32974045 OR 19167144 OR 34986170** | 4/4 |

**Database:** Embase (via Elsevier)
**Search Date**: 9/6/2024

| **Concept** | **Strategy** | **Results** |
| --- | --- | --- |
| #1 Adolescents | adolescent/exp OR 'young adult'/exp OR ('young adult' OR youth OR youths OR adolescent OR adolescents OR teen OR teens OR teenager OR teenagers OR teenaged OR 'high school' OR 'high schools' OR 'high schooler' OR 'high schoolers' OR 'secondary school' OR 'secondary schools' OR 'secondary schooler' OR 'secondary schoolers' OR college OR pubescent OR pubescence OR 'young person' OR 'young persons' OR 'young people' OR puberty):ti,ab NOT ((adult/exp OR infant/exp) NOT adolescent/exp) | 2,354,213 |
| #2 Mental Health | 'mental health'/exp OR 'mental health care'/exp OR 'mental disease'/exp OR suicide/exp OR anxiety/exp OR 'anxiety disorder'/exp OR 'impulse control disorder'/exp OR 'dissociative disorder'/exp OR 'mood disorder'/exp OR 'eating disorder'/exp OR 'depression'/exp OR 'disorders of higher cerebral function'/exp OR 'cognition defect'/exp OR 'schizophrenia spectrum'/exp OR 'behavioral'/exp OR cognition/exp OR dyssomnias/de OR 'posttraumatic stress disorder'/exp OR ((behavior* OR behaviour* OR mental OR mood OR psychologic* OR psychiatric OR neurocogn* OR neuropsychiatric OR neuro-psychological OR neuro-psychiatric OR neuropsychological OR mental OR mentally OR stress* OR affective OR panic* OR phobic OR phobia* OR psychotic OR somatoform) NEAR/3 (health OR disease OR diseases OR disorder OR disorders OR illness OR ill OR symptom OR symptoms OR symptomatic OR syndrome OR syndromes OR ailment OR ailments)):ti,ab OR (suicid* OR depress* OR PTSD OR CPTSD OR anxiety OR anxious):ti,ab | 6,581,581 |
| #3 Peer Support | 'peer group'/exp OR 'group therapy'/exp OR ((peer OR peers) NEAR/2 (group* OR support* OR educat* OR instruct* OR facilitat* OR mentor* OR influenc* OR navigat* OR leader* OR led OR supervis* OR manag* OR relation* OR communicat* OR relationship* OR influence* OR 'relationship* communit*' OR network*)) OR (peer$peer OR 'peer to peer' OR peer-to-peer OR peer-group OR peer-led OR peer-mentor*):ti,ab OR ('group interpersonal psychotherapy' OR 'group therapy' OR group-therapy):ti,ab | 83,838 |
| #4 LMIC Emtree | 'developing country'/exp OR afghanistan/exp OR bangladesh/exp OR benin/exp OR 'burkina faso'/exp OR burundi/exp OR cambodia/exp OR 'central african republic'/exp OR chad/exp OR comoros/exp OR 'democratic republic of the congo'/exp OR eritrea/exp OR ethiopia/exp OR gambia/exp OR guinea/exp OR guinea-bissau/exp OR haiti/exp OR kenya/exp OR 'democratic peoples republic of korea'/exp OR liberia/exp OR madagascar/exp OR malawi/exp OR mali/exp OR mozambique/exp OR myanmar/exp OR nepal/exp OR niger/exp OR rwanda/exp OR 'sierra leone'/exp OR somalia/exp OR tajikistan/exp OR tanzania/exp OR togo/exp OR uganda/exp OR zimbabwe/exp OR armenia/exp OR bhutan/exp OR bolivia/exp OR cameroon/exp OR 'cabo verde'/exp OR congo/exp OR 'cote d ivoire'/exp OR djibouti/exp OR egypt/exp OR 'el salvador'/exp OR 'georgia (republic)'/exp OR ghana/exp OR guatemala/exp OR guyana/exp OR honduras/exp OR indonesia/exp OR india/exp OR kosovo/exp OR kyrgyzstan/exp OR laos/exp OR lesotho/exp OR mauritania/exp OR micronesia/exp OR moldova/exp OR mongolia/exp OR morocco/exp OR nicaragua/exp OR nigeria/exp OR pakistan/exp OR 'papua new guinea'/exp OR paraguay/exp OR philippines/exp OR 'independent state of samoa'/exp OR 'atlantic islands'/exp OR senegal/exp OR melanesia/exp OR 'sri lanka'/exp OR sudan/exp OR 'south sudan'/exp OR eswatini/exp OR syria/exp OR timor-leste/exp OR ukraine/exp OR uzbekistan/exp OR vanuatu/exp OR vietnam/exp OR 'middle east'/exp OR yemen/exp OR zambia/exp OR angola/exp OR albania/exp OR algeria/exp OR 'american samoa'/exp OR argentina/exp OR azerbaijan/exp OR 'republic of belarus'/exp OR belize/exp OR 'bosnia and herzegovina'/exp OR botswana/exp OR brazil/exp OR bulgaria/exp OR china/exp OR colombia/exp OR 'costa rica'/exp OR cuba/exp OR dominica/exp OR 'dominican republic'/exp OR ecuador/exp OR 'equatorial guinea'/exp OR fiji/exp OR gabon/exp OR grenada/exp OR iran/exp OR iraq/exp OR jamaica/exp OR jordan/exp OR kazakhstan/exp OR lebanon/exp OR libya/exp OR 'republic of north macedonia'/exp OR malaysia/exp OR 'indian ocean islands'/exp OR mexico/exp OR montenegro/exp OR namibia/exp OR palau/exp OR panama/exp OR peru/exp OR romania/exp OR russia/exp OR serbia/exp OR seychelles/exp OR 'south africa'/exp OR 'saint lucia'/exp OR 'saint vincent and the grenadines'/exp OR suriname/exp OR thailand/exp OR tonga/exp OR tunisia/exp OR turkey/exp OR turkmenistan/exp OR venezuela/exp | 1,964,990 |
| #5 LMIC Terms + Demonyms | (afghanistan OR afghan* OR afghanistani* OR afghani* OR afghanese OR bangladesh OR bangladeshi* OR benin OR 'edo people' OR 'edo person' OR bini OR 'burkina faso' OR burkinabe* OR burundi OR burundian* OR cambodia OR cambodian* OR 'cabo verde' OR 'cape verdean' OR 'cape verdeans' OR 'cabo verdean' OR 'cabo verdeans' OR 'central african' OR 'central africans' OR chad OR chadian* OR comoros OR comorian* OR congo OR congolese* OR eritrea OR eritrean* OR ethiopia OR ethiopian* OR gambia OR gambian* OR guinea OR guinean* OR 'guinea bissau' OR 'bissau guinean' OR 'bissau guineans' OR haiti OR haitian* OR kenya OR kenyan* OR 'democratic peoples republic of korea' OR 'north korean' OR 'north korenas' OR liberia OR liberian* OR madagascar OR malagasy* OR madagascan* OR malawi OR malawian* OR mali OR malian* OR mozambique OR mozambican* OR myanmar OR burmese*or myanma* OR nepal OR nepali* OR nepalese OR niger OR nigerien* OR rwanda OR rwandan* OR rwandese OR 'sierra leone' OR 'sierra leonean' OR 'sierra leoneans' OR somalia OR somali* OR tajikistan OR tajikstani* OR tajik* OR tanzania OR tanzanian* OR togo OR togolese* OR uganda OR ugandan* OR zimbabwe OR zimbabwean* OR zimbo* OR armenia OR armenian* OR bhutan OR bhutanese OR bolivia OR bolivian* OR cameroon OR cameroonian* OR 'cape verde' OR congo OR 'cote divoire' OR ivorian* OR djibouti OR djiboutian* OR egypt OR egyptian* OR 'el salvador' OR salvadoran* OR georgian* OR ghana OR ghanaian* OR guatemala OR guatemalan* OR guatemalteco* OR guatemalense* OR guyana OR guyanese OR honduras OR honduran* OR indonesia OR indonesian* OR india OR indian* OR kiribati OR gilbertese* OR kosovo OR kosovar* OR kosovan* OR kyrgyzstan OR kyrgyzstani* OR kirgiz OR kirghiz OR kyrgyz OR laos OR laotian* OR lao OR lesotho OR mosotho* OR basotho* OR mauritania OR mauritanian* OR micronesia OR micronesian* OR moldova OR moldovan* OR mongolia OR mongolian* OR morocco OR moroccan* OR nicaragua OR nicaraguan* OR nigeria OR nigerian* OR pakistan OR pakistani* OR 'papua new guinea' OR 'papua new guinean' OR 'papua new guineans' OR paraguay OR paraguayan* OR philippines OR filipin* OR pinoy* OR pinay* OR samoa OR samoan* OR 'atlantic islands' OR 'sao tome' OR 'sao tomean' OR 'sao tomeans' OR santomean* OR principe OR senegal OR senegalese* OR melanesia OR melanesian* OR 'solomon islands' OR 'solomon islander' OR 'solomon islanders' OR 'sri lanka' OR 'sri lankan' OR 'sri lankans' OR sinhalese OR sudan OR sudanese OR swaziland OR swazi* OR liswati* OR eswatini OR syria OR syrian* OR 'east timor' OR 'east timorese' OR 'timor leste' OR timorese OR ukraine OR ukrainian* OR uzbekistan OR uzbekistani* OR vanuatu OR vanuatuan* OR vietnam OR vietnamese* OR 'middle east' OR 'middle eastern' OR 'west bank' OR gaza OR palestinian* OR gazan* OR yemen OR yemeni* OR zambia OR zambian* OR angola OR angolan* OR albania OR albanian* OR algeria OR algerian* OR argentina OR argentine* OR argentinean* OR argentinian* OR samoa OR samoan* OR azerbaijan OR azerbaijani* OR azeri* OR belarus OR belarusian* OR belize OR belizean* OR 'bosnia herzegovina' OR bosnian* OR botswana OR batswana* OR motswana* OR brazil OR brazilian* OR bulgaria OR bulgarian* OR china OR chinese OR colombia OR colombian* OR 'costa rica' OR 'costa rican' OR 'costa ricans' OR cuba OR cuban* OR dominica OR dominican* OR 'dominican republic' OR ecuador OR ecuadorian* OR 'equatorial guinea' OR equatoguinean* OR 'equatorial guinean' OR 'equatorial guineans' OR fiji OR fijian* OR gabon OR gabonese OR gabonaise OR grenada OR grenadian* OR iran OR iranian* OR iraq OR iraqi* OR jamaica OR jamaican* OR jordan OR jordanian* OR kazakhstan OR kazakhstani* OR lebanon OR lebanese OR libya OR libyan* OR macedonia OR macedonian* OR malaysia OR malaysian* OR 'indian ocean islands' OR maldives OR maldivian* OR 'marshall islands' OR marshallese OR mauritius OR mauritian* OR mexico OR mexican* OR montenegro OR montenegrin* OR namibia OR namibian* OR palau OR palauan* OR panama OR panamanian* OR peru OR peruvian* OR romania OR romanian* OR russia OR russian* OR serbia OR serbian* OR seychelles OR seychellois* OR seselwa* OR 'south africa' OR 'south african' OR 'south africans' OR 'saint lucia' OR 'saint lucian' OR 'saint lucians' OR 'st vincent' OR 'saint vincent' OR grenadine OR 'west indies' OR vincentian* OR grenadinian* OR vincy OR suriname OR surinamese* OR thailand OR thai OR tonga OR tongan* OR tunisia OR tunisian* OR turkey OR turkish OR turk OR turkmenistan OR turkmenistani* OR turkmen* OR turkmenian* OR tuvalu OR tuvaluan* OR venezuela OR venezuelan*):ti,ab,kw | 2,479,807 |
| #6 LMIC Additional Terms | ('low resource' OR underresourced OR 'resource poor' OR 'resource limited' OR 'under developed' OR underdeveloped OR 'developing country' OR 'developing countries' OR 'developing world' OR 'third world' OR lmic OR lmics OR 'central America' OR 'central american' OR 'south America' OR 'south American' OR 'southeast Asia' OR 'southeast Asian' OR 'pacific islands' OR 'pacific islander' OR 'pacific islanders' OR 'sub saharan' OR Caribbean OR 'latin America' OR 'latin American' OR oceania):ti,ab,kw OR (low NEAR/2 income):ti,ab,kw OR (middle NEAR/2 income):ti,ab,kw | 421,702 |
| #7 Combined LMIC Filter | #4 OR #5 OR #6 | 3,293,956 |
| #8 Combined | #1 AND #2 AND #3 AND #7 | 2,478 |
| #9 Exclusions | #8 NOT ('editorial'/exp OR [editorial]/lim OR 'note'/exp OR [note]/lim OR [conference abstract]/lim OR 'conference abstract'/exp OR 'conference abstract'/it) | 2,107 |

**Database:** Web of Science (via Clarivate)
**Search Date**: 9/6/2024

| **Concept** | **Strategy** | **Results** |
| --- | --- | --- |
| #1 Adolescents | TS=("young adult" OR youth OR youths OR adolescent OR adolescents OR teen OR teens OR teenager OR teenagers OR teenaged OR "high school" OR "high schools" OR "high schooler" OR "high schoolers" OR "secondary school" OR "secondary schools" OR "secondary schooler" OR "secondary schoolers" OR college OR pubescent OR pubescence OR "young person'" OR "young persons" OR "young people" OR puberty) | 1,315,083 |
| #2 Mental Health | TS=((behavior* OR behaviour* OR mental OR mood OR psychologic* OR psychiatric OR neurocogn* OR neuropsychiatric OR neuro-psychological OR neuro-psychiatric OR neuropsychological OR mental OR mentally OR stress* OR affective OR panic* OR phobic OR phobia* OR psychotic OR somatoform) NEAR/3 (health OR disease OR diseases OR disorder OR disorders OR illness OR ill OR symptom OR symptoms OR symptomatic OR syndrome OR syndromes OR ailment OR ailments)) OR TS=(suicid* OR depress* OR PTSD OR CPTSD OR anxiety OR anxious) | 1,851,380 |
| #3 Peer Support | TS=((peer OR peers) NEAR/2 (group* OR support* OR educat* OR instruct* OR facilitat* OR mentor* OR influenc* OR navigat* OR leader* OR led OR supervis* OR manag* OR relation* OR communicat* OR relationship* OR influence* OR "relationship* communit*" OR network*)) OR TS=(peer?peer OR "peer to peer" OR peer-to-peer OR peer-group OR peer-led OR peer-mentor*) OR TS=("group interpersonal psychotherapy" OR "group therapy" OR group-therapy) | 93,054 |
| #4 LMIC Terms | TS=(afghanistan OR afghan* OR afghanistani* OR afghani* OR afghanese OR bangladesh OR bangladeshi* OR benin OR "edo people" OR "edo person" OR bini OR "burkina faso" OR burkinabe* OR burundi OR burundian* OR cambodia OR cambodian* OR "cabo verde" OR "cape verdean" OR "cape verdeans" OR "cabo verdean" OR "cabo verdeans" OR "central african republic" OR "central african" OR "central africans" OR chad OR chadian* OR comoros OR comorian* OR "democratic republic of the congo" OR congolese* OR eritrea OR eritrean* OR ethiopia OR ethiopian* OR gambia OR gambian* OR guinea OR guinean* OR guinea-bissau OR "bissau guinean" OR "bissau guineans" OR bissau-guinean* OR haiti OR haitian* OR kenya OR kenyan* OR "democratic people's republic of korea" OR "north korean" OR "north korenas" OR liberia OR liberian* OR madagascar OR malagasy* OR madagascan* OR malawi OR malawian* OR mali OR malian* OR mozambique OR mozambican* OR myanmar OR burmese*or myanma* OR nepal OR nepali* OR nepalese OR niger OR nigerien* OR rwanda OR rwandan* OR rwandese OR "sierra leone" OR "sierra leonean" OR "sierra leoneans" OR somalia OR somali* OR tajikistan OR tajikstani* OR tajik* OR tanzania OR tanzanian* OR togo OR togolese* OR uganda OR ugandan* OR zimbabwe OR zimbabwean* OR zimbo* OR armenia OR armenian* OR bhutan OR bhutanese OR bolivia OR bolivian* OR cameroon OR cameroonian* OR "cape verde" OR congo OR "cote d'ivoire" OR ivorian* OR djibouti OR djiboutian* OR egypt OR egyptian* OR "el salvador" OR salvadoran* OR "georgia (republic)" OR georgian* OR ghana OR ghanaian* OR guatemala OR guatemalan* OR guatemalteco* OR guatemalense* OR guyana OR guyanese OR honduras OR honduran* OR indonesia OR indonesian* OR india OR indian* OR kiribati OR gilbertese* OR kosovo OR kosovar* OR kosovan* OR kyrgyzstan OR kyrgyzstani* OR kirgiz OR kirghiz OR kyrgyz OR laos OR laotian* OR lao OR lesotho OR mosotho* OR basotho* OR mauritania OR mauritanian* OR micronesia OR micronesian* OR moldova OR moldovan* OR mongolia OR mongolian* OR morocco OR moroccan* OR nicaragua OR nicaraguan* OR nigeria OR nigerian* OR pakistan OR pakistani* OR "papua new guinea" OR "papua new guinean" OR "papua new guineans" OR paraguay OR paraguayan* OR philippines OR filipin* OR pinoy* OR pinay* OR "independent state of samoa" OR samoan* OR "atlantic islands" OR "sao tome" OR "sao tomean" OR "sao tomeans" OR santomean* OR principe OR senegal OR senegalese* OR melanesia OR melanesian* OR "solomon islands" OR "solomon islander" OR "solomon islanders" OR "sri lanka" OR "sri lankan" OR "sri lankans" OR sinhalese OR sudan OR sudanese OR swaziland OR swazi* OR liswati* OR eswatini OR syria OR syrian* OR "east timor" OR "east timorese" OR "timor leste" OR timorese OR ukraine OR ukrainian* OR uzbekistan OR uzbekistani* OR vanuatu OR vanuatuan* OR vietnam OR vietnamese* OR "middle east" OR "middle eastern" OR "west bank" OR gaza OR palestinian* OR gazan* OR yemen OR yemeni* OR zambia OR zambian* OR angola OR angolan* OR albania OR albanian* OR algeria OR algerian* OR argentina OR argentine* OR argentinean* OR argentinian* OR samoa OR samoan* OR azerbaijan OR azerbaijani* OR azeri* OR "republic of belarus" OR belarus OR belarusian* OR belize OR belizean* OR bosnia-herzegovina OR bosnian* OR botswana OR batswana* OR motswana* OR brazil OR brazilian* OR bulgaria OR bulgarian* OR china OR chinese OR colombia OR colombian* OR "costa rica" OR "costa rican" OR "costa ricans" OR cuba OR cuban* OR dominica OR dominican* OR "dominican republic" OR ecuador OR ecuadorian* OR "equatorial guinea" OR equatoguinean* OR "equatorial guinean" OR "equatorial guineans" OR fiji OR fijian* OR gabon OR gabonese OR gabonaise OR grenada OR grenadian* OR iran OR iranian* OR iraq OR iraqi* OR jamaica OR jamaican* OR jordan OR jordanian* OR kazakhstan OR kazakhstani* OR lebanon OR lebanese OR libya OR libyan* OR macedonia OR macedonian* OR malaysia OR malaysian* OR "indian ocean islands" OR maldives OR maldivian* OR "marshall islands" OR marshallese OR mauritius OR mauritian* OR mexico OR mexican* OR montenegro OR montenegrin* OR namibia OR namibian* OR palau OR palauan* OR panama OR panamanian* OR peru OR peruvian* OR romania OR romanian* OR russia OR russian* OR serbia OR serbian* OR seychelles OR seychellois* OR seselwa* OR "south africa" OR "south african" OR "south africans" OR "saint lucia" OR "saint lucian" OR "saint lucians" OR "saint vincent AND the grenadines"or "st vincent" OR "saint vincent" OR "grenadines" OR "west indies" OR vincentian* OR grenadinian* OR vincy OR suriname OR surinamese* OR thailand OR thai OR tonga OR tongan* OR tunisia OR tunisian* OR turkey OR turkish OR turk OR turkmenistan OR turkmenistani* OR turkmen* OR turkmenian* OR tuvalu OR tuvaluan* OR venezuela OR venezuelan* OR "low resource" OR "under-resourced" OR underresourced OR "resource poor" OR "resource limited" OR "under-developed" OR underdeveloped OR "developing country" OR "developing countries" OR "developing world" OR "third world" OR lmic OR lmics OR "central America" OR "central american" OR "south America" OR "south American" OR "southeast Asia" OR "southeast Asian" OR "pacific islands" OR "pacific islander" OR "pacific islanders" OR sub-saharan OR Caribbean OR "latin America" OR "latin American" OR oceania) OR TS=(low NEAR/2 income) OR TS=(middle NEAR/2 income) | 6,067,127 |
| #5 Combined | #1 AND #2 AND #3 AND #4 | 1,218 |
| #6 Exclusions | Refine by "Document Type: Article; Review Article | 1,197 |

**Database:** Global Health (via EBSCO)
**Search Date**: 9/6/2024

| **Concept** | **Strategy** | **Results** |
| --- | --- | --- |
| S1 Adolescents | (TI "young adult" OR TI youth OR TI youths OR TI adolescent OR TI adolescents OR TI teen OR TI teens OR TI teenager OR TI teenagers OR TI teenaged OR TI "high school" OR TI "high schools" OR TI "high schooler" OR TI "high schoolers" OR TI "secondary school" OR TI "secondary schools" OR TI "secondary schooler" OR TI "secondary schoolers" OR TI college OR TI pubescent OR TI pubescence OR TI "young person'" OR TI "young persons" OR TI "young people" OR TI puberty) OR (AB "young adult" OR AB youth OR AB youths OR AB adolescent OR AB adolescents OR AB teen OR AB teens OR AB teenager OR AB teenagers OR AB teenaged OR AB "high school" OR AB "high schools" OR AB "high schooler" OR AB "high schoolers" OR AB "secondary school" OR AB "secondary schools" OR AB "secondary schooler" OR AB "secondary schoolers" OR AB college OR AB pubescent OR AB pubescence OR AB "young person'" OR AB "young persons" OR AB "young people" OR AB puberty) | 166,300 |
| S2 Mental Health | ((TI behavior* OR TI behaviour* OR TI mental OR TI mood OR TI psychologic* OR TI psychiatric OR TI neurocogn* OR TI neuropsychiatric OR TI neuro-psychological OR TI neuro-psychiatric OR TI neuropsychological OR TI mental OR TI mentally OR TI stress* OR TI affective OR TI panic* OR TI phobic OR TI phobia* OR TI psychotic OR TI somatoform) N3 (health OR TI disease OR TI diseases OR TI disorder OR TI disorders OR TI illness OR TI ill OR TI symptom OR TI symptoms OR TI symptomatic OR TI syndrome OR TI syndromes OR TI ailment OR TI ailments)) OR (TI suicid* OR TI depress* OR TI PTSD OR TI CPTSD OR TI anxiety OR TI anxious) OR ((AB behavior* OR AB behaviour* OR AB mental OR AB mood OR AB psychologic* OR AB psychiatric OR AB neurocogn* OR AB neuropsychiatric OR AB neuro-psychological OR AB neuro-psychiatric OR AB neuropsychological OR AB mental OR AB mentally OR AB stress* OR AB affective OR AB panic* OR AB phobic OR AB phobia* OR AB psychotic OR AB somatoform) N3 (health OR AB disease OR AB diseases OR AB disorder OR AB disorders OR AB illness OR AB ill OR AB symptom OR AB symptoms OR AB symptomatic OR AB syndrome OR AB syndromes OR AB ailment OR AB ailments)) OR (AB suicid* OR AB depress* OR AB PTSD OR AB CPTSD OR AB anxiety OR AB anxious) | 189,488 |
| S3 Peer Support | ((TI peer OR TI peers) N2 (group* OR TI support* OR TI educat* OR TI instruct* OR TI facilitat* OR TI mentor* OR TI influenc* OR TI navigat* OR TI leader* OR TI led OR TI supervis* OR TI manag* OR TI relation* OR TI communicat* OR TI relationship* OR TI influence* OR TI "relationship* communit*" OR TI network*)) OR (TI peer?peer OR TI "peer to peer" OR TI peer-to-peer OR TI peer-group OR TI peer-led OR TI peer-mentor*) OR (TI "group interpersonal psychotherapy" OR TI "group therapy" OR TI group-therapy) OR ((AB peer OR AB peers) N2 (group* OR AB support* OR AB educat* OR AB instruct* OR AB facilitat* OR AB mentor* OR AB influenc* OR AB navigat* OR AB leader* OR AB led OR AB supervis* OR AB manag* OR AB relation* OR AB communicat* OR AB relationship* OR AB influence* OR AB "relationship* communit*" OR AB network*)) OR (AB peer?peer OR AB "peer to peer" OR AB peer-to-peer OR AB peer-group OR AB peer-led OR AB peer-mentor*) OR (AB "group interpersonal psychotherapy" OR AB "group therapy" OR AB group-therapy) | 7,722 |
| S4 LMIC Terms | ((TI afghanistan OR AB afghanistan) OR (TI afghan* OR AB afghan*) OR (TI afghanistani* OR AB afghanistani*) OR (TI afghani* OR AB afghani*) OR (TI afghanese OR AB afghanese) OR (TI bangladesh OR AB bangladesh) OR (TI bangladeshi* OR AB bangladeshi*) OR (TI benin OR AB benin) OR (TI "edo people" OR AB "edo people") OR (TI "edo person" OR AB "edo person") OR (TI bini OR AB bini) OR (TI "burkina faso" OR AB "burkina faso") OR (TI burkinabe* OR AB burkinabe*) OR (TI burundi OR AB burundi) OR (TI burundian* OR AB burundian*) OR (TI cambodia OR AB cambodia) OR (TI cambodian* OR AB cambodian*) OR (TI "cabo verde" OR AB "cabo verde") OR (TI "cape verdean" OR AB "cape verdean") OR (TI "cape verdeans" OR AB "cape verdeans") OR (TI "cabo verdean" OR AB "cabo verdean") OR (TI "cabo verdeans" OR AB "cabo verdeans") OR (TI "central african republic" OR AB "central african republic") OR (TI "central african" OR AB "central african") OR (TI "central africans" OR AB "central africans") OR (TI chad OR AB chad) OR (TI chadian* OR AB chadian*) OR (TI comoros OR AB comoros) OR (TI comorian* OR AB comorian*) OR (TI "democratic republic of the congo" OR AB "democratic republic of the congo") OR (TI congolese* OR AB congolese*) OR (TI eritrea OR AB eritrea) OR (TI eritrean* OR AB eritrean*) OR (TI ethiopia OR AB ethiopia) OR (TI ethiopian* OR AB ethiopian*) OR (TI gambia OR AB gambia) OR (TI gambian* OR AB gambian*) OR (TI guinea OR AB guinea) OR (TI guinean* OR AB guinean*) OR (TI guinea-bissau OR AB guinea-bissau) OR (TI "bissau guinean" OR AB "bissau guinean") OR (TI "bissau guineans" OR AB "bissau guineans") OR (TI bissau-guinean* OR AB bissau-guinean*) OR (TI haiti OR AB haiti) OR (TI haitian* OR AB haitian*) OR (TI kenya OR AB kenya) OR (TI kenyan* OR AB kenyan*) OR (TI "democratic people's republic of korea" OR AB "democratic people's republic of korea") OR (TI "north korean" OR AB "north korean") OR (TI "north korenas" OR AB "north korenas") OR (TI liberia OR AB liberia) OR (TI liberian* OR AB liberian*) OR (TI madagascar OR AB madagascar) OR (TI malagasy* OR AB malagasy*) OR (TI madagascan* OR AB madagascan*) OR (TI malawi OR AB malawi) OR (TI malawian* OR AB malawian*) OR (TI mali OR AB mali) OR (TI malian* OR AB malian*) OR (TI mozambique OR AB mozambique) OR (TI mozambican* OR AB mozambican*) OR (TI myanmar OR AB myanmar) OR (TI "burmese*or myanma*" OR AB "burmese*or myanma*") OR (TI nepal OR AB nepal) OR (TI nepali* OR AB nepali*) OR (TI nepalese OR AB nepalese) OR (TI niger OR AB niger) OR (TI nigerien* OR AB nigerien*) OR (TI rwanda OR AB rwanda) OR (TI rwandan* OR AB rwandan*) OR (TI rwandese OR AB rwandese) OR (TI "sierra leone" OR AB "sierra leone") OR (TI "sierra leonean" OR AB "sierra leonean") OR (TI "sierra leoneans" OR AB "sierra leoneans") OR (TI somalia OR AB somalia) OR (TI somali* OR AB somali*) OR (TI tajikistan OR AB tajikistan) OR (TI tajikstani* OR AB tajikstani*) OR (TI tajik* OR AB tajik*) OR (TI tanzania OR AB tanzania) OR (TI tanzanian* OR AB tanzanian*) OR (TI togo OR AB togo) OR (TI togolese* OR AB togolese*) OR (TI uganda OR AB uganda) OR (TI ugandan* OR AB ugandan*) OR (TI zimbabwe OR AB zimbabwe) OR (TI zimbabwean* OR AB zimbabwean*) OR (TI zimbo* OR AB zimbo*) OR (TI armenia OR AB armenia) OR (TI armenian* OR AB armenian*) OR (TI bhutan OR AB bhutan) OR (TI bhutanese OR AB bhutanese) OR (TI bolivia OR AB bolivia) OR (TI bolivian* OR AB bolivian*) OR (TI cameroon OR AB cameroon) OR (TI cameroonian* OR AB cameroonian*) OR (TI "cape verde" OR AB "cape verde") OR (TI congo OR AB congo) OR (TI "cote d'ivoire" OR AB "cote d'ivoire") OR (TI ivorian* OR AB ivorian*) OR (TI djibouti OR AB djibouti) OR (TI djiboutian* OR AB djiboutian*) OR (TI egypt OR AB egypt) OR (TI egyptian* OR AB egyptian*) OR (TI "el salvador" OR AB "el salvador") OR (TI salvadoran* OR AB salvadoran*) OR (TI "georgia (republic)" OR AB "georgia (republic)") OR (TI georgian* OR AB georgian*) OR (TI ghana OR AB ghana) OR (TI ghanaian* OR AB ghanaian*) OR (TI guatemala OR AB guatemala) OR (TI guatemalan* OR AB guatemalan*) OR (TI guatemalteco* OR AB guatemalteco*) OR (TI guatemalense* OR AB guatemalense*) OR (TI guyana OR AB guyana) OR (TI guyanese OR AB guyanese) OR (TI honduras OR AB honduras) OR (TI honduran* OR AB honduran*) OR (TI indonesia OR AB indonesia) OR (TI indonesian* OR AB indonesian*) OR (TI india OR AB india) OR (TI indian* OR AB indian*) OR (TI kiribati OR AB kiribati) OR (TI gilbertese* OR AB gilbertese*) OR (TI kosovo OR AB kosovo) OR (TI kosovar* OR AB kosovar*) OR (TI kosovan* OR AB kosovan*) OR (TI kyrgyzstan OR AB kyrgyzstan) OR (TI kyrgyzstani* OR AB kyrgyzstani*) OR (TI kirgiz OR AB kirgiz) OR (TI kirghiz OR AB kirghiz) OR (TI kyrgyz OR AB kyrgyz) OR (TI laos OR AB laos) OR (TI laotian* OR AB laotian*) OR (TI lao OR AB lao) OR (TI lesotho OR AB lesotho) OR (TI mosotho* OR AB mosotho*) OR (TI basotho* OR AB basotho*) OR (TI mauritania OR AB mauritania) OR (TI mauritanian* OR AB mauritanian*) OR (TI micronesia OR AB micronesia) OR (TI micronesian* OR AB micronesian*) OR (TI moldova OR AB moldova) OR (TI moldovan* OR AB moldovan*) OR (TI mongolia OR AB mongolia) OR (TI mongolian* OR AB mongolian*) OR (TI morocco OR AB morocco) OR (TI moroccan* OR AB moroccan*) OR (TI nicaragua OR AB nicaragua) OR (TI nicaraguan* OR AB nicaraguan*) OR (TI nigeria OR AB nigeria) OR (TI nigerian* OR AB nigerian*) OR (TI pakistan OR AB pakistan) OR (TI pakistani* OR AB pakistani*) OR (TI "papua new guinea" OR AB "papua new guinea") OR (TI "papua new guinean" OR AB "papua new guinean") OR (TI "papua new guineans" OR AB "papua new guineans") OR (TI paraguay OR AB paraguay) OR (TI paraguayan* OR AB paraguayan*) OR (TI philippines OR AB philippines) OR (TI filipin* OR AB filipin*) OR (TI pinoy* OR AB pinoy*) OR (TI pinay* OR AB pinay*) OR (TI "independent state of samoa" OR AB "independent state of samoa") OR (TI samoan* OR AB samoan*) OR (TI "atlantic islands" OR AB "atlantic islands") OR (TI "sao tome" OR AB "sao tome") OR (TI "sao tomean" OR AB "sao tomean") OR (TI "sao tomeans" OR AB "sao tomeans") OR (TI santomean* OR AB santomean*) OR (TI principe OR AB principe) OR (TI senegal OR AB senegal) OR (TI senegalese* OR AB senegalese*) OR (TI melanesia OR AB melanesia) OR (TI melanesian* OR AB melanesian*) OR (TI "solomon islands" OR AB "solomon islands") OR (TI "solomon islander" OR AB "solomon islander") OR (TI "solomon islanders" OR AB "solomon islanders") OR (TI "sri lanka" OR AB "sri lanka") OR (TI "sri lankan" OR AB "sri lankan") OR (TI "sri lankans" OR AB "sri lankans") OR (TI sinhalese OR AB sinhalese) OR (TI sudan OR AB sudan) OR (TI sudanese OR AB sudanese) OR (TI swaziland OR AB swaziland) OR (TI swazi* OR AB swazi*) OR (TI liswati* OR AB liswati*) OR (TI eswatini OR AB eswatini) OR (TI syria OR AB syria) OR (TI syrian* OR AB syrian*) OR (TI "east timor" OR AB "east timor") OR (TI "east timorese" OR AB "east timorese") OR (TI "timor leste" OR AB "timor leste") OR (TI timorese OR AB timorese) OR (TI ukraine OR AB ukraine) OR (TI ukrainian* OR AB ukrainian*) OR (TI uzbekistan OR AB uzbekistan) OR (TI uzbekistani* OR AB uzbekistani*) OR (TI vanuatu OR AB vanuatu) OR (TI vanuatuan* OR AB vanuatuan*) OR (TI vietnam OR AB vietnam) OR (TI vietnamese* OR AB vietnamese*) OR (TI "middle east" OR AB "middle east") OR (TI "middle eastern" OR AB "middle eastern") OR (TI "west bank" OR AB "west bank") OR (TI gaza OR AB gaza) OR (TI palestinian* OR AB palestinian*) OR (TI gazan* OR AB gazan*) OR (TI yemen OR AB yemen) OR (TI yemeni* OR AB yemeni*) OR (TI zambia OR AB zambia) OR (TI zambian* OR AB zambian*) OR (TI angola OR AB angola) OR (TI angolan* OR AB angolan*) OR (TI albania OR AB albania) OR (TI albanian* OR AB albanian*) OR (TI algeria OR AB algeria) OR (TI algerian* OR AB algerian*) OR (TI argentina OR AB argentina) OR (TI argentine* OR AB argentine*) OR (TI argentinean* OR AB argentinean*) OR (TI argentinian* OR AB argentinian*) OR (TI samoa OR AB samoa) OR (TI samoan* OR AB samoan*) OR (TI azerbaijan OR AB azerbaijan) OR (TI azerbaijani* OR AB azerbaijani*) OR (TI azeri* OR AB azeri*) OR (TI "republic of belarus" OR AB "republic of belarus") OR (TI belarus OR AB belarus) OR (TI belarusian* OR AB belarusian*) OR (TI belize OR AB belize) OR (TI belizean* OR AB belizean*) OR (TI bosnia-herzegovina OR AB bosnia-herzegovina) OR (TI bosnian* OR AB bosnian*) OR (TI botswana OR AB botswana) OR (TI batswana* OR AB batswana*) OR (TI motswana* OR AB motswana*) OR (TI brazil OR AB brazil) OR (TI brazilian* OR AB brazilian*) OR (TI bulgaria OR AB bulgaria) OR (TI bulgarian* OR AB bulgarian*) OR (TI china OR AB china) OR (TI chinese OR AB chinese) OR (TI colombia OR AB colombia) OR (TI colombian* OR AB colombian*) OR (TI "costa rica" OR AB "costa rica") OR (TI "costa rican" OR AB "costa rican") OR (TI "costa ricans" OR AB "costa ricans") OR (TI cuba OR AB cuba) OR (TI cuban* OR AB cuban*) OR (TI dominica OR AB dominica) OR (TI dominican* OR AB dominican*) OR (TI "dominican republic" OR AB "dominican republic") OR (TI ecuador OR AB ecuador) OR (TI ecuadorian* OR AB ecuadorian*) OR (TI "equatorial guinea" OR AB "equatorial guinea") OR (TI equatoguinean* OR AB equatoguinean*) OR (TI "equatorial guinean" OR AB "equatorial guinean") OR (TI "equatorial guineans" OR AB "equatorial guineans") OR (TI fiji OR AB fiji) OR (TI fijian* OR AB fijian*) OR (TI gabon OR AB gabon) OR (TI gabonese OR AB gabonese) OR (TI gabonaise OR AB gabonaise) OR (TI grenada OR AB grenada) OR (TI grenadian* OR AB grenadian*) OR (TI iran OR AB iran) OR (TI iranian* OR AB iranian*) OR (TI iraq OR AB iraq) OR (TI iraqi* OR AB iraqi*) OR (TI jamaica OR AB jamaica) OR (TI jamaican* OR AB jamaican*) OR (TI jordan OR AB jordan) OR (TI jordanian* OR AB jordanian*) OR (TI kazakhstan OR AB kazakhstan) OR (TI kazakhstani* OR AB kazakhstani*) OR (TI lebanon OR AB lebanon) OR (TI lebanese OR AB lebanese) OR (TI libya OR AB libya) OR (TI libyan* OR AB libyan*) OR (TI macedonia OR AB macedonia) OR (TI macedonian* OR AB macedonian*) OR (TI malaysia OR AB malaysia) OR (TI malaysian* OR AB malaysian*) OR (TI "indian ocean islands" OR AB "indian ocean islands") OR (TI maldives OR AB maldives) OR (TI maldivian* OR AB maldivian*) OR (TI "marshall islands" OR AB "marshall islands") OR (TI marshallese OR AB marshallese) OR (TI mauritius OR AB mauritius) OR (TI mauritian* OR AB mauritian*) OR (TI mexico OR AB mexico) OR (TI mexican* OR AB mexican*) OR (TI montenegro OR AB montenegro) OR (TI montenegrin* OR AB montenegrin*) OR (TI namibia OR AB namibia) OR (TI namibian* OR AB namibian*) OR (TI palau OR AB palau) OR (TI palauan* OR AB palauan*) OR (TI panama OR AB panama) OR (TI panamanian* OR AB panamanian*) OR (TI peru OR AB peru) OR (TI peruvian* OR AB peruvian*) OR (TI romania OR AB romania) OR (TI romanian* OR AB romanian*) OR (TI russia OR AB russia) OR (TI russian* OR AB russian*) OR (TI serbia OR AB serbia) OR (TI serbian* OR AB serbian*) OR (TI seychelles OR AB seychelles) OR (TI seychellois* OR AB seychellois*) OR (TI seselwa* OR AB seselwa*) OR (TI "south africa" OR AB "south africa") OR (TI "south african" OR AB "south african") OR (TI "south africans" OR AB "south africans") OR (TI "saint lucia" OR AB "saint lucia") OR (TI "saint lucian" OR AB "saint lucian") OR (TI "saint lucians" OR AB "saint lucians") OR (TI "saint vincent and the grenadinesor "st vincent"" OR AB "saint vincent and the grenadinesor "st vincent"") OR (TI "saint vincent" OR AB "saint vincent") OR (TI grenadines OR AB grenadines) OR (TI "west indies" OR AB "west indies") OR (TI vincentian* OR AB vincentian*) OR (TI grenadinian* OR AB grenadinian*) OR (TI vincy OR AB vincy) OR (TI suriname OR AB suriname) OR (TI surinamese* OR AB surinamese*) OR (TI thailand OR AB thailand) OR (TI thai OR AB thai) OR (TI tonga OR AB tonga) OR (TI tongan* OR AB tongan*) OR (TI tunisia OR AB tunisia) OR (TI tunisian* OR AB tunisian*) OR (TI turkey OR AB turkey) OR (TI turkish OR AB turkish) OR (TI turk OR AB turk) OR (TI turkmenistan OR AB turkmenistan) OR (TI turkmenistani* OR AB turkmenistani*) OR (TI turkmen* OR AB turkmen*) OR (TI turkmenian* OR AB turkmenian*) OR (TI tuvalu OR AB tuvalu) OR (TI tuvaluan* OR AB tuvaluan*) OR (TI venezuela OR AB venezuela) OR (TI venezuelan* OR AB venezuelan*) OR (TI "low resource" OR AB "low resource") OR (TI under-resourced OR AB under-resourced) OR (TI underresourced OR AB underresourced) OR (TI "resource poor" OR AB "resource poor") OR (TI "resource limited" OR AB "resource limited") OR (TI under-developed OR AB under-developed) OR (TI underdeveloped OR AB underdeveloped) OR (TI "developing country" OR AB "developing country") OR (TI "developing countries" OR AB "developing countries") OR (TI "developing world" OR AB "developing world") OR (TI "third world" OR AB "third world") OR (TI lmic OR AB lmic) OR (TI lmics OR AB lmics) OR (TI "central America" OR AB "central America") OR (TI "central american" OR AB "central american") OR (TI "south America" OR AB "south America") OR (TI "south American" OR AB "south American") OR (TI "southeast Asia" OR AB "southeast Asia") OR (TI "southeast Asian" OR AB "southeast Asian") OR (TI "pacific islands" OR AB "pacific islands") OR (TI "pacific islander" OR AB "pacific islander") OR (TI "pacific islanders" OR AB "pacific islanders") OR (TI sub-saharan OR AB sub-saharan) OR (TI Caribbean OR AB Caribbean) OR (TI "latin America" OR AB "latin America") OR (TI "latin American" OR AB "latin American") OR (TI oceania OR AB oceania)) OR ((TI low OR AB low) N2 (TI income OR AB income)) OR ((TI middle OR AB middle) N2 (TI income OR AB income)) | 1,208,080 |
| S5 Combined | S1 AND S2 AND S3 AND S4 | 240 |
| S6 Exclusions | Limit "Source Types" to "Academic Journals" | 235 |

**Database: Global Index Medicus (via WHO)**
Search date: 9/6/2024
*Note: all indicies including AIM, LILACS, IMEMR, IMSEAR, WPRIM; use Advanced Search and set to search Title, abstract, subject*

| **Concept** | **Strategy** | **Results** |
| --- | --- | --- |
| #1 Adolescents | mh:(adolescents OR "young adults") OR tw:(young adult or youth or youths or adolescent or adolescents or teen or teens or teenager or teenagers or teenaged or "high school" or "high schools" or "high schooler" or "high schoolers" or "secondary school" or "secondary schools" or "secondary schooler" or "secondary schoolers" or college or pubescent or pubescence or "young person" or "young persons" or  "young people" or puberty) | 188,482 |
| #2 Mental Health | mh:("mental health" or "mental disorders" or suicide or anxiety or "anxiety disorders" or "disruptive, impulse control, and conduct disorders" or "dissociative disorders" or "mood disorders" or "depressive disorder" or "neurocognitive disorders" or "cognition disorders" or  "schizophrenia spectrum and other psychotic disorders" or "behavioral symptoms" or cognition dyssomnias or "stress disorders, post-traumatic") or tw:((behavior* or behaviour* or mental or mood or psychologic* or "psychiatric or neurocogn*" or neuropsychiatric or neuro-psychological or neuro-psychiatric or neuropsychological or mental or mentally or stress* or affective or panic* or phobic or phobia* or psychotic or somatoform) AND (health or disease or diseases or disorder or disorders or illness or ill or symptom or symptoms or symptomatic or syndrome or syndromes or ailment or ailments)) or tw:(suicid* or depress* or PTSD or CPTSD or anxiety or anxious) | 227,031 |
| #3 Peer Support | tw:((peer or peers) AND (group* or support* or educat* or instruct* or facilitat* or mentor* or influenc* or navigat* or leader* or led or supervis* or manag* or relation* or communicat* or relationship* or influence* or relationship* communit* or network*)) | 4,597 |
| #$ Combined | #1 AND #2 AND #3 | 544 |
